# Supplementary material for: Polymorphisms in Pfkelch13 domains before and after the introduction of artemisinin-based combination therapy in Southwest Nigeria
Source: PLoS One. 2025 Mar 31;20(3):e0316479. doi: 10.1371/journal.pone.0316479 (PMC11957316; doi:10.1371/journal.pone.0316479)

Samples: 12665
Bases: 1057
Average spacing: 12

8/17/2022

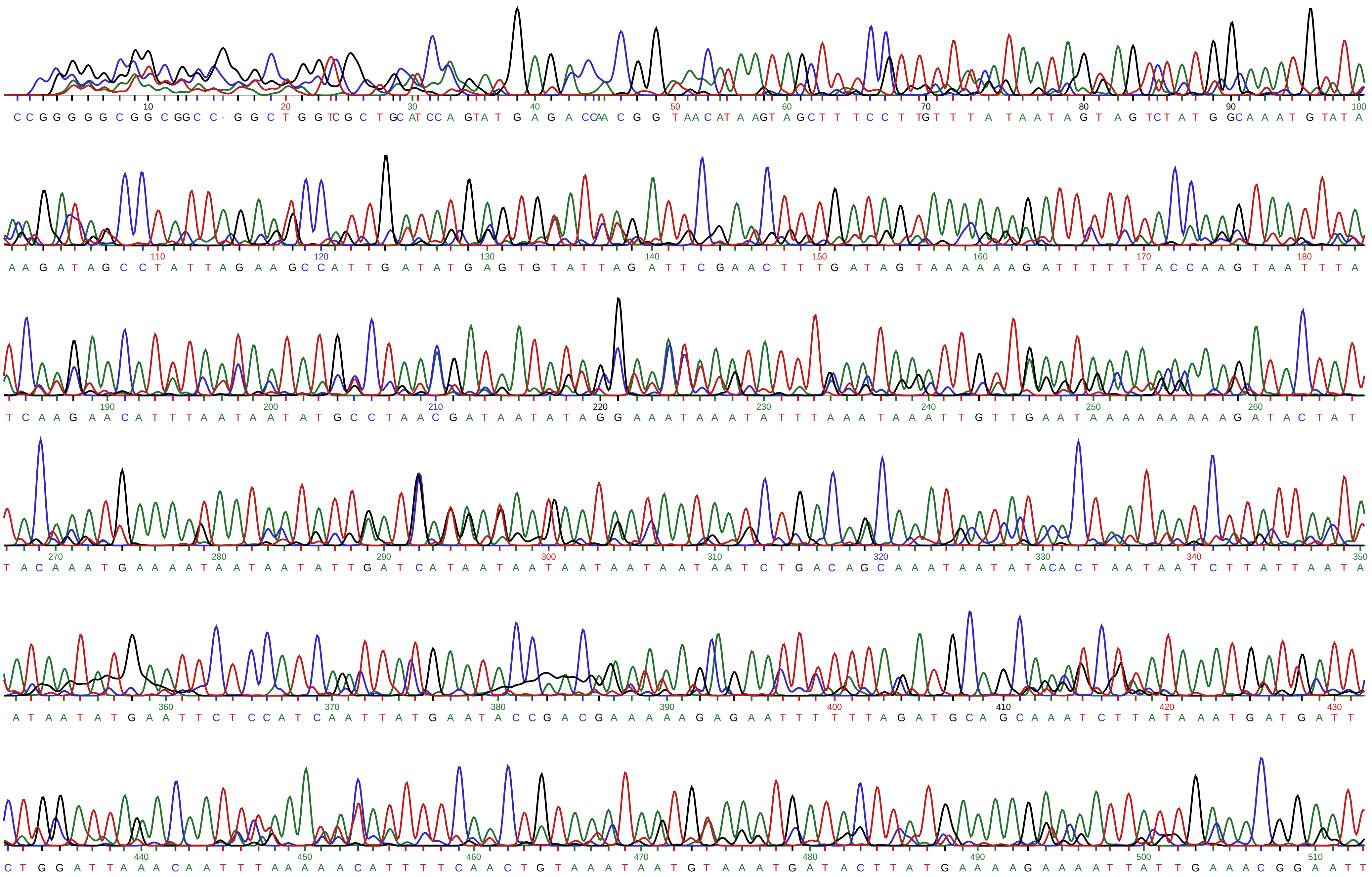

Samples: 12665
Bases: 1057
Average spacing: 12

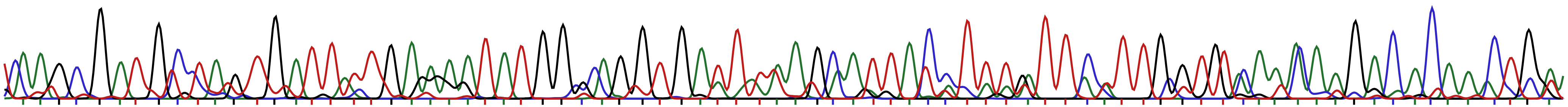

A A G C G A T G C T A G T G A T T T T G A A A A T A T G G C A G G T G A T T T A A G C A T T A C C T T T A T T C A T T G G T T C A A A A A G A C A C A A C T G A

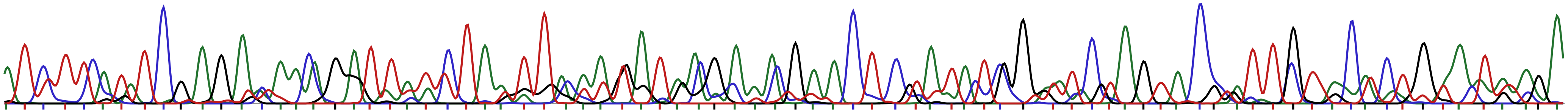

A T C T C A T T C G A G A C A A A G A T T A T C T A A T T A A A T A T A A G A A A G A A C T C T A T A T C G T T C T A G T A C G A T T G T A C A C T G T A T A A G A

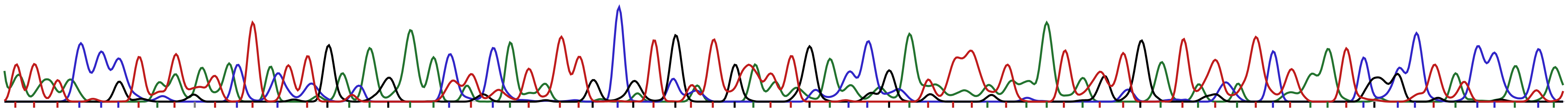

T T T C C C T A T - T C T C T G A A G A A C T C A T T T G C G T G T T G A T T G A C C G A T T T A T A A T A T T G A T A T A T C T A A T C G C C T A C C A C A

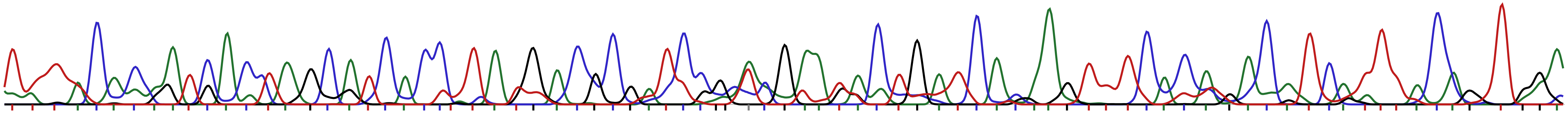

T T T A C A C A A T C A C T A G C A T C A C C T T A T G A C G C G A T C C G G - C G A A T A C T G A T C A C A A G T T T C A C A G A C A T C A T T T A C A G T G G A

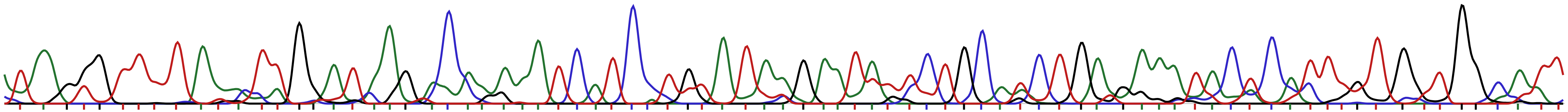

T A G T G T T T T A T A T T G G A T A A G A C A A A A A T C A T C C T G T A T A A G A A T A T T C T G C A T C T G A G A A A T A C T C A T T T G T G T T G G C A T T

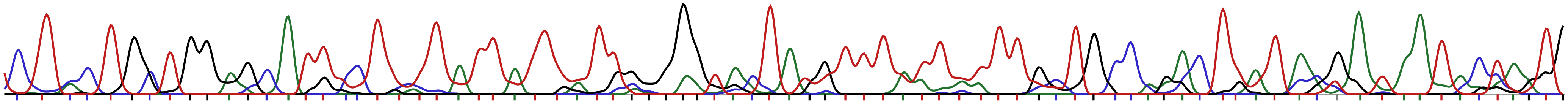

C T C C T G C T G G A G C A T T C C T T T T A T T A T T A T T G G G G G T A C T A G G T T T A T T T T T T G C T G C C A G A C T T A T A C - A T A A T A C T A G T G

Samples: 12665
Bases: 1057
Average spacing: 12

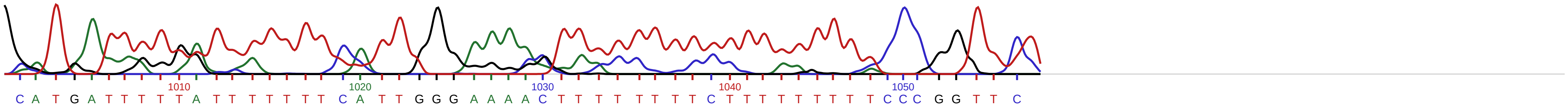

Supplement: Supporting information 2 — (ZIP) [file pone.0316479.s002.zip › 056KN2F_PREMIX_Plate_KELCH2_A10.pdf]
